# Reply to: “Sleep quality is influenced by multiple factors and cannot be reduced to the volume of pineal gland calcification”

**DOI:** 10.1055/s-0046-1824434

**Published:** 2026-06-25

**Authors:** Oscar H. Del Brutto, Robertino M. Mera, Vishal Patel, Pablo R. Castillo

**Affiliations:** 1 Universidad Espíritu Santo, Escuela de Medicina, Samborondón, Ecuador.; 2 Universidad Espíritu Santo, Centro de Investigaciones, Samborondón, Ecuador.; 3 Freenome Inc., Department of Biostatistics/Epidemiology, South San Francisco CA, United States.; 4 Mayo Clinic, College of Medicine & Science, Department of Radiology, Jacksonville FL, United States.; 5 Mayo Clinic, College of Medicine & Science, Sleep Disorders Center, Jacksonville FL, United States.

Dear Editor,

We appreciate the interest of Scorza et al. 1 In our article, 2 and welcome the opportunity to clarify several points raised in their letter. We agree that sleep quality is multifactorial. However, our study did not aim to model all determinants of sleep quality. Instead, our objective was to assess the association between automated measurements of pineal gland calcification (PGC) volumes and sleep quality after adjusting for age and sex. This approach is consistent with prior epidemiological work exploring the relationship between pineal morphology and sleep parameters. Importantly, our population is unusually homogeneous in lifestyle, socioeconomic status, diet, and environmental exposure, as we explicitly described. Many of the external factors listed by Scorza et al (noise, light pollution, shift work, socioeconomic variability etc.) are minimal or absent in this setting, which is precisely why this population is ideal for studying biological correlates of sleep.

We acknowledge the limitations of subjective sleep questionnaires. However, the Pittsburgh Sleep Quality Index (PSQI) is one of the most widely used tools in sleep epidemiology. 3 It has been translated, validated, and successfully used in previous studies. 4 5 Scorza et al. cites limitations observed in Chinese professional athletes, which is a population with different characteristics from rural Ecuadorian villagers. 6 The PSQI's performance is known to vary by context, which is precisely why local validation is essential.

While polysomnography (PSG) is the gold standard for sleep architecture, it is not feasible in large population–based field studies in rural settings. This is a common and accepted limitation in epidemiology. Our aim was not to diagnose sleep disorders but to evaluate self–reported sleep quality, which is the construct measured by the PSQI. 1 Scorza et al. suggest that subjective assessments are misleading, yet subjective sleep quality is a clinically meaningful outcome and is widely used in public health research.

Age is a major determinant of PGC, which is why we adjusted for it. Scorza et al. enumerated additional factors, but many are either extremely rare in our population, not measurable in a population–based field study, or unlikely to confound the association given the population's homogeneity. Moreover, the authors' suggestion that all factors influencing PGC must be included in the model is statistically unrealistic and unnecessary. Epidemiological modeling requires adjustment for major confounders, not an exhaustive list of all possible correlates.

The comment regarding the PSQI item on sleep medication use also reflects a misunderstanding of the instrument's purpose. This questionnaire does not classify types of sleep medications; it simply captures their use, which is standard in PSQI–based research. Illicit drug use is exceedingly uncommon in our population, further reducing the likelihood of this confounding factor.

The assertion that the algorithm used to calculate PGC volume was not specified is incorrect. Our article provides a detailed description of the method: brain structures were segmented using SynthSeg (Alzheimer's Disease Neuroimaging Initiative), a spherical pineal region of interest was defined, voxels exceeding 50 HU were considered calcified, and an expert neuroradiologist validated the region of interest in 50 randomly sampled cases. This level of methodological transparency exceeds that of most prior studies on PGC.

Finally, the claim that all factors influencing pineal gland size and PSQI must be included in the multivariate model is neither statistically feasible nor scientifically necessary. Our analysis appropriately adjusted for the major confounders relevant to the study question, and the central finding remains unchanged: after adjusting for age and sex, PGC volume is not associated with sleep quality in this population. This conclusion is robust, consistent with our prior work, and contributes meaningfully to the ongoing discussion regarding the functional significance of pineal calcification.
